# Respiratory and nonrespiratory COVID-19 complications in patients with obesity: recent developments

**DOI:** 10.2217/cer-2021-0237

**Published:** 2022-01-13

**Authors:** Esther Liu, Hudson Lee, Briana Lui, Robert S White, Jon D Samuels

**Affiliations:** ^1^Weill Cornell Medical College, Weill Cornell Medicine, 1300 York Ave, New York, NY 10065, USA; ^2^Department of Anesthesiology, Weill Cornell Medicine, 525 East 68th Street, Box 124, New York, NY 10065, USA

**Keywords:** BMI, complications, COVID-19, obesity

## Abstract

This narrative review summarizes recent reports to provide an updated understanding of the multiorgan effects of SARS-CoV-2 infection in obese individuals. A PubMed search of 528 primary articles was performed, with inclusion based on novelty, relevance and redundancy. Obesity confers an increased risk for hospitalization, intensive care unit admission, severe pneumonia, intubation and acute kidney injury in COVID-19 patients. Obesity is also associated with higher levels of inflammatory and thrombotic markers. However, the associations between obesity and mortality or cardiac injury in COVID-19 patients remain unclear. Obesity is a risk factor for several respiratory and nonrespiratory COVID-19 complications. Future work is needed to further explore these relationships and optimize the management of obese COVID-19 patients.

## Background

The initial cases of coronavirus disease 2019 (COVID-19), caused by severe acute respiratory syndrome coronavirus 2 (SARS-CoV-2), were first reported in Wuhan, China, and have since grown into a global pandemic. As of 5 September 2021, globally there were more than 220 million total confirmed cases of SARS-CoV-2 infection and over 4.7 million COVID-related deaths [1]. Emerging evidence suggests that obesity and aspects of impaired metabolic health such as hyperglycemia, hypertension, subclinical inflammation and various comorbidities, such as diabetes and cardiovascular disease, are associated with a high risk of developing severe COVID-19 [2,3]. Given the obesity epidemic in the USA, there has been particular interest in research focusing on the mechanisms of SARS-CoV-2 infection in obese individuals as well as their health outcomes.

There are several potential mechanisms for the increased susceptibility of obese individuals to COVID-19 infection and subsequent complications. SARS-CoV-2 infects human cells in the lung and other tissues by binding to angiotensin-converting enzyme 2 (ACE2) receptors present on the plasma membrane [4]. ACE2 receptor expression is increased in adipose tissue compared with that in the lung; thus, obese individuals with more adipose tissue have increased ACE2 receptor expression, which facilitates the entry of SARS-CoV-2 into adipocytes [5]. As a result, adipose tissue serves as a reservoir for the virus, and may facilitate its spread to surrounding organs [6,7]. Additionally, a recent study by Shin *et al.* [8] found that the cell surface GRP78, which is highly expressed in adipocytes, facilitates the binding and accumulation of SARS-CoV-2 in ACE2-expressing cells. The overexpression of GRP78 was found to be associated with hyperinsulinemia in adipocytes, mediated by stress-responsive transcription factor XBP-1s. This study highlights the role of GRP78 as a binding partner of the SARS-CoV-2 spike protein and ACE2, which may contribute to the pathogenesis of severe progression and severe outcomes of COVID-19 in obese patients. Of note, metabolically healthy obesity exists and is not associated with a large increase in cardiometabolic risk [9,10]. This may be explained by the body fat distribution and adipocyte biology or function in the specific white adipose tissue fat depots of these individuals.

Given their increased susceptibility to SARS-CoV-2 infection, it is critical to understand the outcomes and complications of infection in obese individuals. There has been extensive work examining the association between obesity and the severity of COVID-19 or respiratory complications. Compared with general obesity, abdominal or visceral obesity may contribute more to the clinical deterioration associated with SARS-CoV-2 infection via compromised pulmonary function (decreased diaphragmatic excursion), impaired immune function and chronic inflammation [11]. Several studies suggest that obesity is strongly associated with an increased risk for hospitalization, intensive care unit (ICU) admission, and the need for invasive mechanical ventilation (IMV) [12–14]. The relationship between obesity and mortality or nonrespiratory complications such as renal or cardiovascular function, however, are less clear. As a result, this review aims to synthesize existing literature on obesity and COVID-19 complications in order to provide an updated view of the multiorgan effects of SARS-CoV-2 infection in obese patients. Specifically, we aim to highlight the following COVID-19 outcomes: hospitalization and ICU admission, mortality, pneumonia and acute respiratory distress syndrome (ARDS), IMV, inflammation and vascular effects, kidney injury and cardiac injury.

## Hospitalization & ICU admission

Numerous studies have found that obesity is associated with a higher risk of hospitalization in COVID-19 patients, and that this relationship is nonlinear, whereby the risk for hospitalization increases with higher BMI [15–20]. Obesity is also associated with a faster time to hospitalization in patients with community-managed COVID-19 pneumonia. Table 1 lists studies as well as their characteristics, outcome, main findings and limitations.

**Table 1.** A summary of notable available studies on obesity and COVID-19 outcomes.

Study (year)	Study design	Country	Sample size (n)	Defined obesity	Defined comparator	COVID-19 outcome	Findings	Limitations	Ref.
Al-Salameh *et al.* (2021)	Retrospective cohort	France	433	BMI ≥25 kg/m^2^	BMI <25 kg/m^2^	ICU admission or death	Overweight and obesity had higher risk of ICU admission	Missing BMI data	[15]
Anderson *et al.* (2020)	Retrospective cohort	USA	2466	WHO criteria^†^	Overweight (BMI 25–29.9 kg/m^2^)	Intubation or death	Obesity was associated with higher risk of intubation or death in patients <65 years old	Missing BMI data for 28% of cohort Small subgroup sizes in stratified analyses	[11]
Cai *et al.* (2020)	Prospective	China	383	BMI ≥28 kg/m^2^	BMI 18.5–23.9 kg/m^2^	Progression to severe COVID-19	Overweight and obese patients had higher risk of developing severe COVID-19	Small sample size, single geographic location Reduced comparability of findings due to defined BMI categories	[21]
Cottini *et al.* (2021)	Prospective	Italy	338	BMI ≥30 kg/m^2^	BMI <25 kg/m^2^	Time to hospitalization	Obese patients had faster time to hospitalization	Limited availability of confirmatory COVID-19 testing	[14]
Dana *et al.* (2021)	Retrospective cohort	France	222	Mild to moderate obesity (30–39.9 kg/m^2^) Severe obesity (≥40 kg/m^2^)	BMI 18.5–24.9 kg/m^2^	In-hospital mortality	No increase in mortality in moderately obese patients	Small sample size	[22]
Frank *et al.* (2020)	Retrospective cohort	USA	305	BMI ≥30 kg/m^2^	BMI <25 kg/m^2^	Intubation or death	Obese patients had increased risk of intubation or death	Single geographic location No long-term follow-up	[23]
Goyal *et al.* (2020)	Retrospective cohort	USA	1687	WHO criteria^†^	BMI 18.5–24.9 kg/m^2^	Respiratory failure (need for IMV) and in-hospital mortality	Obesity was an independent risk factor for respiratory failure	Sample from single geographic location	[24]
Hajifathalian *et al.* (2020)	Retrospective cohort	USA	770	BMI ≥30 kg/m^2^	BMI ≥18.5 and <30	ICU admission or death	Obesity was associated with higher risk of ICU admission or death	No control group Lack of power for analyzing BMI extremes	[16]
Hendren *et al.* (2020)	Retrospective cohort	USA	8519	WHO criteria^†^	18.5–24.9 kg/m^2^	In-hospital death, mechanical ventilation, dialysis, venous thromboembolism, cardiac events	Obesity was associated with higher risk of in-hospital mortality, IMV, venous thromboembolism and dialysis	No formal adjudication of nonfatal outcomes	[25]
Jayanama *et al.* (2021)	Retrospective cohort	Thailand	147	≥25.0 kg/m^2^	18.5–22.9 kg/m^2^	Severe pneumonia, AKI, ICU stay	Obesity was associated with severe pneumonia and AKI, transaminitis and length of ICU stay	Small sample size	[20]
Kompaniyets *et al.* (2021)	Retrospective cohort	USA	148,494	WHO criteria^†^ Class III further subdivided into 40–44.9 kg/m^2^ group and ≥45 kg/m^2^ group	BMI 18.5–24.9 kg/m^2^	Hospitalization, ICU admission, IMV, mortality	A dose-response relationship was found between BMI and hospitalization, ICU admission, IMV and death	Missing BMI data Risk estimates measured only for patients receiving care at hospital	[13]
McNeill *et al.* (2021)	Retrospective cohort	USA	781	BMI ≥30 kg/m^2^	BMI <30 kg/m^2^	Inflammatory markers: CRP, ESR, IL-6, D-dimer, ferritin	Obesity was associated with greater inflammation (higher initial and peak CRP, ESR and peak D-dimer)	Inflammatory markers were not measured at set intervals Single center study	[26]
Monteiro *et al.* (2020)	Retrospective cohort	USA	112	Not available	Not available	Need for IMV	Obesity and smoking were risk factors for the need for IMV	Small sample size, single location Short follow-up BMI information not included	[9]
Motaib *et al.* (2021)	Retrospective cohort	Pakistan	107	BMI ≥30 kg/m^2^	BMI 18.5–24.9 kg/m^2^	ICU admission	Obesity was associated with ICU admission	Small sample size	[19]
Nakeshbandi *et al.* (2020)	Retrospective cohort	USA	684	BMI ≥30 kg/m^2^	BMI 18.50–24.99 kg/m^2^	30 day in-hospital mortality, intubation, AKI, ARDS, acute cardiac injury	Obesity was associated with increased risk of intubation	Predominantly African-American sample, limiting generalizability	[27]
Nyabera *et al.* (2020)	Retrospective cohort	USA	290	BMI >30 kg/m^2^	BMI 18.5–30 kg/m^2^	Mortality	BMI was not found to be an independent predictor of mortality	Small sample size	[28]
Onder *et al.* (2020)	Retrospective cohort	Italy	3694	Not available	Not available	Acute renal failure, shock	Obesity was associated with increased risk of acute renal failure and shock, especially in younger patients	Data on BMI not available	[25]
Simonnet *et al.* (2020)	Retrospective cohort	France	124	BMI >30 kg/m^2^	BMI 18.5 to <25 kg/m^2^	Need for IMV	The proportion of patients requiring IMV increased with BMI	Small sample size	[8]
Singh *et al.* (2020)	Retrospective cohort	USA	41,513	BMI of ≥30 kg/m^2^ or diagnosis of obesity	BMI of <30 kg/m^2^ or no reported diagnosis of obesity	Mortality, intubation or hospitalization	Obese COVID-19 patients had higher risk of hospitalization, intubation or death	Possible errors in coding from EHR database	[12]
Steinberg *et al.* (2020)	Retrospective cohort	USA	210	BMI >30 kg/m^2^	BMI = <30 kg/m^2^	Hospitalization, need for IMV, in-hospital mortality	There was a significant association between obesity and hospitalization, need for IMV and in-hospital mortality	Sample size from small geographic area Comorbid conditions unaccounted for	[7]
Ullah *et al.* (2021)	Retrospective cohort	USA	176	WHO criteria^†^	BMI 18.5–24.9 kg/m^2^	Need for IMV, dialysis, upgrade to ICU, mortality	Patients with higher BMIs had higher risk of in-hospital mortality	Small sample size	[29]
Wu *et al.* (2021)	Retrospective cohort	China	1091	BMI ≥25 kg/m^2^	BMI 18.5–23 kg/m^2^	IMV, ICU admission, ARDS, death	Obese patients had increased risk of IMV, ICU admission and ARDS	Reduced comparability of findings due to defined BMI categories	[18]
Zhang *et al.* (2020)	Retrospective cohort	China	53	≥28.0 kg/m^2^	18.5–23.9 kg/m^2^	Mortality, cardiac injury, coagulation activity	Obesity was a risk factor for high mortality, mediated by cardiac injury and coagulation activity	Small sample size	[30]

† WHO criteria: underweight (<18.5 kg/m^2^), normal weight (18.5–24.9 kg/m^2^), overweight (25.0–29.9 kg/m^2^), class 1 obesity (30–34.9 kg/m^2^), class 2 obesity (35–39.9 kg/m^2^) and class 3 obesity (≥40 kg/m^2^).

AKI: Acute kidney injury; ARDS: Acute respiratory distress syndrome; CRP: C-reactive protein; EHR: Electronic health record; ESR: Erythrocyte sedimentation rate; ICU: Intensive care unit; IMV: Invasive mechanical ventilation.

Steinberg *et al.* [15] conducted a retrospective cohort study with 210 younger adult patients (18–45 years old) who tested positive for SARS-CoV-2 between March and April 2020. Admission was significantly associated with a BMI over 30 kg/m^2^ (odds ratio [OR]: 2.61; 95% CI: 1.49–4.58; p = .0008). The authors concluded that obesity is an independent risk factor for hospitalization in young obese patients with COVID-19. Limitations include the small sample size and the fact that comorbidities that may have impacted the clinical course of COVID-19 were not taken into consideration. Additionally, Singh *et al.* [18] conducted a retrospective cohort study with a large nationally representative database of 41,513 adult COVID-19 patients in the USA and found that patients with any degree of obesity (BMI >30 kg/m^2^) had a significantly higher risk of hospitalization compared with patients without obesity.

Kompaniyets *et al.* [19] found that among 148,494 adults with COVID-19 at US hospitals between March and December 2020, BMI was a risk factor for hospitalization, forming a J-shaped nonlinear curve. The risk for hospitalization was lowest among patients with a BMI of 24.2 kg/m^2^, defined as a healthy weight, and increased markedly with higher BMIs. This dose-response relationship was pronounced for patients over 65 years old, consistent with previous studies [16,17]. A recent study by Cottini *et al.* [20] found that obesity was associated with a higher hospitalization risk and a shorter interval from infection to hospitalization in patients in Bergamo, Italy with community-managed COVID-19 pneumonia. Obese patients were more likely to require hospitalization than nonobese patients (hazard ratio [HR]: 5.83; 95% CI: 3.91–8.71). The findings in this study may not translate to other ethnic and racial groups, and deserve additional investigation.

Interestingly, Kerrigan *et al.* [31] demonstrated that cardiorespiratory fitness can attenuate the impact of risk factors for COVID-19-related hospitalization. In 246 patients who were tested positive for SARS-CoV-2, obesity was not associated with an increased hospital risk, contradicting the findings of previous studies. However, when cardiorespiratory fitness was introduced via a clinically indicated exercise stress test (between January 2016 and February 2020), obesity had a paradoxical protective effect. This may be explained by the cohort composition of this study, which mainly consisted of patients physically able to perform exercise stress tests on a treadmill. Additionally, more fit individuals may have had a higher BMI due to increased muscle mass, rather than increased adipose tissue, highlighting a limitation of using BMI as a sole measure of obesity. Indeed, although BMI is well suited for population-based studies, BMI is only an estimate of adiposity and is unable to account for body fat distribution or distinguish between lean muscle versus fat mass [29,31].

Several studies have also shown that obesity is a risk factor for ICU admission [19,21,22,24,28,32,33]. A meta-analysis published early in the COVID-19 pandemic found that obesity was associated with a 39% increased risk of developing critical illness, which included ICU admission [34]. Further, Hajifathalian *et al.* [32] performed a retrospective review of 770 adult patients with COVID-19 between March and April 2020 at two hospitals in New York City. Obese patients (BMI >30 kg/m^2^) had nearly twice the risk of critical illness leading to ICU admission compared with normal weight patients after adjusting for confounders such as age, race and troponin level (risk ratio (RR): 1.76; p: 0.001). Although another study by Sattar *et al.* [35] found a higher risk of ICU admission specifically in obese patients <60 years old, Hajifathalian *et al.* [32] found this association to hold for all age groups. However, as Hajifathalian *et al.* did not include a control group, these findings require external validation to ensure generalizability to other populations.

Though obese patients appear to have a higher risk of ICU admission, recent studies disagree on whether this also applies to overweight individuals [21,24]. Al-Salameh *et al.* [24] retrospectively assessed 433 patients with COVID-19 from Amiens University Hospital in France, and found that overweight status – and not only obesity – is associated with ICU admission. Relative to patients with BMI <25 kg/m^2^, patients who were overweight or obese had a higher risk of being admitted to the ICU, with ORs (95% CI) of 3.16 (1.29–8.06) and 3.05 (1.25–7.82), respectively. It is important to note that several patients were missing BMI data, which may have affected the observed patterns. However, these results conflict with a study by Motaib *et al.* [21], which found that being overweight was not associated with ICU admission. The authors assessed 107 patients with confirmed COVID-19 between March and May 2020, and found that only obesity was associated with ICU admission (OR: 9.11; 95% CI: 1.49–55.84), though the results are limited by the small sample size. Future studies are needed to clarify the relationship between overweight status and ICU admission.

## Mortality

There is conflicting evidence regarding whether obesity is associated with a higher risk of COVID-19-related mortality. Ullah *et al.* [36] conducted a retrospective cohort study of 176 patients with confirmed COVID-19 between March and May 2020. Patients with higher BMIs had a higher risk of in-hospital mortality (OR: 3.2; 95% CI: 1.3–8.2; p = 0.01) after adjusting for medication use and baseline comorbidities. Cottini *et al.* [20] also found that obese patients with BMI ≥30 kg/m^2^ had a higher risk of mortality (p < 0.05) and time-to-death (p = 0.184) compared with normal weight patients (BMI: 18.5–24.9 kg/m^2^).

Further, Kompaniyets *et al.* [19] found that BMI was associated with mortality in a dose-dependent manner both for all ages and split into age <65 years or ≥65 years. The J-shaped mortality versus BMI curve suggests that obese patients of all ages with BMI ranging from 30–34.9 kg/m^2^ (adjusted RR 1.08; 95% CI: 1.02–1.14) to >45 kg/m^2^ (adjusted RR: 1.61; 95% CI: 1.47–1.76) have higher risk of mortality compared with patients who are underweight (<18.5 kg/m^2^) or normal weight (18.5–24.9 kg/m^2^). After adjusting for other underlying comorbidities with a sensitivity analysis, the authors found weaker associations between BMI and severe COVID-19-associated illness. This finding may be due to the inclusion of intermediate variables in the relationship between BMI and COVID-19 outcome, such as cardiovascular disease, hypertension, diabetes and cancers. Despite the large sample size, 28% of patients across the 238 included hospitals were missing height or weight information, precluding inclusion in the study. A recent study by Huang *et al.* [37] suggests that this J-shaped relationship – which highlights an association between overweight (20–25 kg/m^2^) and lower mortality – may be attributable to issues with reverse causation and the presence of confounders.

However, some recent studies suggest that obesity does not increase the risk for COVID-19-related mortality. Goyal *et al.* [27] did not find an association between BMI and in-hospital mortality for 1687 hospitalized patients with COVID-19 at two hospitals in New York City. Specifically, overweight status with BMI 25–29.9 kg/m^2^ (adjusted HR [aHR]: 0.75; 95% CI: 0.56–1.00), mild to moderate obesity with BMI 30–39.9 kg/m^2^ (aHR: 0.98; 95% CI: 0.70–1.36), and morbid obesity with BMI ≥40 kg/m^2^ (aHR: 1.41; 95% CI: 0.74–2.70) were not associated with increased mortality risk compared with reference BMI (18.5–24.9 kg/m^2^). Wu *et al.* [22] performed a retrospective cohort study of 1091 patients at two hospitals in Wuhan, China between January and March 2020, and found that neither overweight status (BMI: 23–25 kg/m^2^) nor obesity (≥25 kg/m^2^) – defined according to WHO recommendations for Asian populations – were found to be significantly associated with mortality compared with normal weight patients (BMI: 18.5–23 kg/m^2^) [38]. Similarly, Nyabera *et al.* [23] found that out of 290 patients older than 65 years admitted to a New York City hospital with COVID-19 between February and April 2020 (reference BMI: 18.5–30 kg/m^2^), BMI was not significantly associated with mortality in ranges from less than 18.5 kg/m^2^ (OR: 0.48; 95% CI: 0.11–2.06; p = 0.32) to over 40 kg/m^2^ (OR: 0.50; 95% CI: 0.13–1.85; p = 0.30).

One recent study provided evidence for a possible obesity paradox for COVID-19 patients in the ICU. Dana *et al.* [39] performed a retrospective cohort study of 222 ICU patients at a hospital in Nancy, France from February to April 2020. Interestingly, patients with moderate obesity of BMI 30–39.9 kg/m^2^ had the lowest in-hospital mortality rate of 13.8%, compared with 17.6% for normal weight (BMI: 18.5–24.9 kg/m^2^), 21.7% for overweight (BMI: 25–29.9 kg/m^2^) and 50% for severe obesity (BMI: ≥40 kg/m^2^). A multivariable logistic regression revealed there was a significantly higher mortality risk among COVID-19 patients who were normal weight or overweight (OR: 3.64; 95% CI: 1.38–9.60) or severely obese (OR: 10.45; 95% CI: 2.45–41.09) compared with patients who were moderately obese. This lends support toward a possible obesity paradox for COVID-19 mortality with ICU patients, but more studies are necessary to corroborate these findings.

## Pneumonia & ARDS

Obesity increases the risk for developing severe pneumonia in patients with COVID-19 [22,26,40,41]. Cai *et al.* [40] performed a study of 383 COVID-19 adult patients admitted to a hospital in Shenzhen, China between January and February 2020. Patients who were overweight (24.0–27.9 kg/m^2^) or obese (≥28 kg/m^2^) had 1.86- and 2.42-fold higher odds, respectively, of developing severe pneumonia. Additionally, overweight men (OR: 1.96; 95% CI: 0.78–4.98) and obese men (OR: 5.70; 95% CI: 1.83– 17.76) had higher risk than overweight women (OR: 1.51; 95% CI: 0.57–4.01) and obese women (OR: 0.71; 95% CI: 0.07–7.30) for developing severe pneumonia. Overall, obese patients were more likely to progress to severe pneumonia due to COVID-19 infection and to exhibit typical symptoms of upper respiratory tract infection. These findings are consistent with previous studies demonstrating that excessive weight gain may increase the risk of developing community-acquired pneumonia [25,30].

Several possible explanations exist for an increased risk of severe COVID-19 pneumonia in the obese patient population, such as altered respiratory mechanisms, reduced forced vital capacity, increased airway resistance and inability to exchange gas effectively [4,22,41,42]. Furthermore, given the increased abdominal pressure on the thorax, the lungs have reduced capacity to expand, leading to ventilation-perfusion mismatch and hypoxemia. Obese patients may also exhibit a pro-inflammatory state as part of metabolic syndrome or a delayed/weakened immune response, thereby facilitating SARS-CoV-2 infection and contributing to lung injury.

ARDS is one of the many possible outcomes of severe COVID-19-induced pneumonia [22,26,41,43]. There is conflicting evidence regarding whether obesity is associated with ARDS in the context of COVID-19 [22,26]. Wu *et al.* [22] found a linear relationship between BMI and likelihood of ARDS (p < 0.0001), where obese patients had increased risk of developing ARDS (HR: 3.15; 95% CI: 1.69–5.88) compared with normal weight patients [22]. However, Nakeshbandi *et al.* [43] examined 684 COVID-19 hospitalized patients between March and April 2020 in New York City and did not find an association between obesity and ARDS. Given these discrepancies, future work is needed to clarify the relationship between obesity and ARDS as an outcome of severe COVID-19 pneumonia.

## Intubation/IMV

Obesity is associated with a greater risk of intubation in COVID-19 patients [17,18,20,28,43–45]. Frank *et al.* [44] conducted a retrospective cohort study of 305 hospitalized COVID-19 patients admitted between March and April 2020. Obese patients had a 2.3-fold increased risk of intubation or death (95% CI: 1.2–4.3) compared with normal weight individuals. Patients with a higher BMI also had more respiratory distress on initial presentation, characterized by dyspnea, requirement for supplemental oxygen and high respiratory rate, which is consistent with prior studies [46,47]. Although this study includes a small sample size from a single geographic region, the results were supported by those of Anderson *et al.* [17], who studied a retrospective cohort of 2466 hospitalized COVID-19 patients in New York City. In this study, obese patients were found to have a higher risk of intubation or death independent of age, sex, race/ethnicity or comorbidities. Patients with class III obesity (≥40 kg/m^2^) had the highest risk (HR: 1.6; 95% CI: 1.1–2.1). Of note, the association between obesity and intubation was only observed in adults under age 65, suggesting that the prevalence of this outcome may differ between younger and older adults.

Several other studies similarly show that obesity is associated with an increased need for IMV [12,15,22,26,36,41,46]. Simonnet *et al.* [46] conducted a retrospective cohort study of 124 consecutive COVID-19 patients admitted to the ICU in a single French medical center between February and April 2020. The proportion of patients requiring IMV increased gradually with BMI, and was highest in patients with a BMI over 35 kg/m^2^, reaching 90% even after adjusting for confounders such as age, diabetes and hypertension. A study by Monteiro *et al.* [12] further supports these findings. In a retrospective observational cohort study of 112 patients with COVID-19 admitted between March and April 2020, obesity was associated with IMV after adjusting for age, sex and comorbidities by multivariable analysis (OR: 5.82; 95% CI: 1.74–19.48). Goyal *et al.* [27] found that obesity was an independent risk factor for respiratory failure and may explain the widespread use of IMV in the USA with patients experiencing severe COVID-19 infection.

## Inflammation & vascular effects

In addition to the well-known respiratory complications of COVID-19, several other complications involving various organ systems have been reported in obese patients, including inflammation, thrombosis and shock. Table 2 highlights recent studies examining these and other rarer, less characterized COVID-19 complications. Obese patients are known to have higher levels of inflammatory and thrombotic markers including C-reactive protein (CRP), erythrocyte sedimentation rate (ESR), peripheral venous blood lymphocyte levels, IL-6 and D-dimer. These effects are exacerbated in the setting of COVID-19, leading to severe disease and death [13,14,48,49].

**Table 2.** Selected studies on rarer COVID-19 complications.

Study (year)	Country	Sample size (n =)	Defined obesity	Defined comparator	Rare COVID-19 complication	Ref.
Hendren *et al.* (2020)	USA	8519	WHO criteria^†^	18.5–24.9 kg/m^2^	Dialysis, venous thromboembolism, cardiac events	[25]
Jayanama *et al.* (2021)	Thailand	147	≥25.0 kg/m^2^	18.5–22.9 kg/m^2^	AKI	[20]
McNeill *et al.* (2021)	USA	781	BMI ≥30 kg/m^2^	BMI <30 kg/m^2^	Inflammatory markers: CRP, ESR, IL-6, D-dimer, ferritin	[26]
Nakeshbandi *et al.* (2020)	USA	684	BMI ≥30 kg/m^2^	BMI 18.50–24.99 kg/m^2^	AKI, acute cardiac injury	[27]
Onder *et al.* (2020)	Italy	3694	Not available	Not available	Acute renal failure, shock	[25]
Ullah *et al.* (2021)	USA	176	WHO criteria^†^	BMI 18.5–24.9 kg/m^2^	Dialysis	[29]
Zhang *et al.* (2020)	China	53	≥28.0 kg/m^2^	18.5–23.9 kg/m^2^	Cardiac injury, coagulation activity	[30]

† WHO criteria: underweight (<18.5 kg/m^2^), normal weight (18.5–24.9 kg/m^2^), overweight (25.0–29.9 kg/m^2^), class 1 obesity (30–34.9 kg/m2), class 2 obesity (35–39.9 kg/m^2^) and class 3 obesity (≥40 kg/m^2^).

AKI: Acute kidney injury; ARDS: Acute respiratory distress syndrome; CRP: C-reactive protein; ESR: Erythrocyte sedimentation rate.

McNeill *et al.* [49] performed a retrospective observational study of 781 hospitalized COVID-19 patients admitted between February and April 2020 to Massachusetts General Hospital. Obese patients with a BMI over 30 kg/m^2^ had significantly elevated peak CRP (147 vs 127 mg/l; p < 0.001), ESR (62 vs 52 mm/h; p = 0.002) and D-dimer (1785 vs 1570 ng/ml; p = 0.004). CRP and ESR are key indices of inflammation, while D-dimer is an indicator of fibrinolysis, coagulation and thrombosis. These associations remained significant after adjusting for age, sex, smoking history and comorbidities such as hypertension (HTN), diabetes mellitus type 2 (DM2), liver disease, kidney disease and pulmonary disease. However, in this study, inflammatory markers were not recorded at set intervals. Mechanistic studies are necessary to better understand the relationship of inflammation and clinical severity of COVID-19 in the obese patient population.

Recent studies suggest that obesity may also be associated with greater risk of venous thromboembolism in patients hospitalized with COVID-19. Hendren *et al.* [50] performed a retrospective analysis of COVID-19 patient data from 7606 patients at 88 US hospitals enrolled in the American Heart Association’s COVID-19 Cardiovascular Disease Registry, with data collected through July 2020. Multivariate Cox models demonstrated that hospitalized COVID-19 patients with class II obesity (BMI: 35.0–39.9 kg/m^2^) had higher risk of deep vein thrombosis (DVT) or pulmonary embolism (PE) (adjusted HR: 1.91; 95% CI: 1.22–2.98) after adjusting for age, sex, race/ethnicity and comorbidities such as CVD, HTN, DM2 and CKD. Interestingly, this effect was not observed for patients with class I (BMI 30–34.9 kg/m^2^) or class III (BMI ≥40 kg/m^2^) obesity.

Shock is another complication of COVID-19 that may be exacerbated by obesity. Onder *et al.* [51] analyzed the medical charts of 3694 patients who died from COVID-19 from a national surveillance system from the Italian National Institute of Health, with data including up to July 2020. They found that shock was associated with obesity in patients who died from COVID-19 (OR: 1.54; 95% CI: 1.19–1.99). This effect was more pronounced in patients under age 60 (OR: 2.37; 95% CI: 1.29–4.36). Of note, BMI data were not available from the database for this study, and surviving COVID-19 patients were not included, potentially limiting the generalizability of results.

## Acute kidney injury

Obesity is associated with albuminuria, kidney disease and decreased glomerular filtration rate [51]. The chronic inflammatory state in obese individuals can trigger inflammatory cascades that precipitate acute kidney failure (AKI) [51]. The deposition of ectopic fat in organs including the liver and kidney has also been linked to reduced organ function and increased clinical severity of COVID-19 [52]. Current research on obesity and renal outcomes in COVID-19 patients suggests that obesity increases the risk for AKI and dialysis [33,41,50,51]. Onder *et al.* [51] found that obesity was significantly associated with increased risk of experiencing AKI (OR: 1.33; 95% CI: 1.04–1.71), and this effect was more pronounced in patients under age 60 (OR: 2.00; 95% CI: 1.29–4.36).

Jayanama *et al.* [33] also found that obesity was associated with AKI in their cohort study of 147 adult COVID-19 patients from Thailand. Obese COVID-19 patients had a higher rate of AKI after adjusting for age, sex, diabetes, hypertension and dyslipidemia. The pathogenesis underlying AKI in obese COVID-19 patients may involve various mechanisms including prerenal azotemia, acute tubular necrosis, direct viral injury, thrombotic microangiopathy and obesity-related inflammation [53]. Additionally, several outcomes such as pneumonia, ARDS and IMV – which may result from COVID-19 infection – may predispose patients to AKI. Consistent with this, several studies have also demonstrated that obese COVID-19 patients are at an increased risk for dialysis [41,50].

## Cardiac injury

Obesity has been linked to cardiac and metabolic diseases such as diabetes and hypertension [33,50,54,55]. Additionally, obesity has been shown to increase the risk of cardiovascular influenza-related complications, leading to increased mortality risk following viral infection [14]. There is conflicting evidence regarding whether there is an association between obesity and cardiac injury in COVID-19 patients [14,17,43,50].

Zhang *et al.* [14] characterized COVID-19 mortality risk in a young obese patient population 14–45 years old. Compared with survivors, deceased patients had a higher BMI (OR: 1.354; 95% CI: 1.075–1.704; p = 0.010) and higher levels of high-sensitive cardiac troponin I, a cardiac injury biomarker (OR: 1.420; 95% CI: 1.112–1.814; p = 0.005). N-terminal probrain natriuretic peptide, another indicator of myocardial damage, was also elevated in deceased patients (OR: 147.5; p = 0.008). Based on these findings, enhanced cardiac injury may be a potential mechanism by which obesity confers higher mortality risk in young COVID-19 patients.

Conversely, there are conflicting studies in the literature that have found that BMI is not associated with cardiac injury in COVID-19 [17,43,50]. Hendren *et al.* [50] found that among 7,606 patients, BMI was not a risk factor for major adverse cardiac events such as heart failure or myocardial infarction even after adjusting for age. Anderson *et al.* [17] and Nakeshbandi *et al.* [43] assessed troponin levels as a marker of cardiac injury, and also did not find an association between obesity and cardiac injury in COVID-19 patients. The discrepancies in findings may be due to differences in the cardiac injury biomarkers that were assessed. Future work is needed to clarify the relationship between obesity and cardiac injury in COVID-19 patients.

## Conclusion & future perspective

The present COVID-19 pandemic has been, and continues to be, a severe cause of disease and death worldwide. It has disproportionately affected patients with various comorbidities that worsen the course of the disease, one of which is obesity. Especially given the high prevalence of obesity in the USA and globally, it is important to continue investigating the impact of obesity on COVID-19 severity, mortality and complications – especially the less-studied inflammatory, vascular, renal and cardiac complications in addition to respiratory effects. Despite this, the findings suggest that multidisciplinary care, often delivered in tertiary care referral medical centers, is required to optimize management of these patients with respect to these complications in order to prevent lasting organ damage, prolonged hospitalization or death as a result of COVID-19 infection. Finally, continuing research is necessary to fully characterize the impact that increased BMI has on these various obese subgroups regarding outcomes and complications, with the ultimate goal of optimizing care of the obese COVID-19 patient during acute and chronic exacerbations. This work will enable a greater understanding of the pathogenic mechanisms underlying COVID-19 as well as its specific respiratory and nonrespiratory mechanisms – ultimately to inform best practices in management to minimize morbidity and mortality, as well as in the development of targeted diagnostics and therapeutics to address the most significant manifestations of COVID-19 and other coronavirus diseases in patients with obesity.

**Executive summary**

- In addition to the well-characterized respiratory complications of COVID-19, there are several nonrespiratory manifestations of COVID-19 that may be exacerbated in obese patients.
- BMI is nonlinearly associated with increased risk of hospitalization, faster time to hospitalization and increased risk of intensive care unit admission in patients with COVID-19.
- There is conflicting evidence regarding whether obesity is associated with increased COVID-19 mortality, but an obesity paradox is unlikely.
- Obesity is associated with increased risk of severe pneumonia as well as the need for invasive mechanical ventilation in COVID-19 patients, but it is unclear if obese COVID-19 patients have an increased risk of acute respiratory distress syndrome.
- COVID-19 patients with obesity are more likely to have increased inflammatory markers, risk of thrombosis and risk of shock.
- Obesity is associated with acute kidney injury and the need for dialysis in the setting of COVID-19.
- It is unclear whether obese patients with COVID-19 have increased markers of cardiac injury or increased risk of major cardiac events.
